# CAR-macrophages: a new chapter in cancer immunotherapy

**DOI:** 10.3724/abbs.2026017

**Published:** 2026-02-11

**Authors:** Xucai Tang, Qian Xiao

**Affiliations:** 1 Institute of Modern Biology Nanjing University Nanjing 210008 China; 2 The State Key Laboratory of Pharmaceutical Biotechnology Nanjing University Nanjing 210008 China; 3 The Affiliated Drum Tower Hospital of Nanjing University Medical School Nanjing University Nanjing 210008 China

**Keywords:** CAR-macrophage (CAR-M), solid tumor, tumor microenvironment remodeling, adoptive cell therapy

## Abstract

Chimeric antigen receptor T (CAR-T) cell therapy achieves remarkable success in hematological cancers, but its efficacy is severely limited in solid tumors by formidable obstacles including physical barriers, the highly immunosuppressive tumor microenvironment (TME), and antigen escape. To address these persistent challenges, chimeric antigen receptor-macrophage (CAR-M) therapy emerges as a promising alternative, leveraging intrinsic advantages of macrophages like unparalleled tumor infiltration, powerful phagocytosis, and high plasticity. The evolution of CAR-M is primarily defined by the intracellular signaling domain. CAR-M exerts its anti-tumor effects through multifaceted mechanisms, including direct enhanced phagocytosis and tumor cell killing, TME remodeling by repolarizing to a pro-inflammatory M1-like phenotype, releasing anti-tumor effectors, and degrading the extracellular matrix (ECM), and the activation of adaptive immunity *via* efficient antigen presentation. Despite its promise, CAR-M faces hurdles such as TME physical barriers and the potential for M2-like re-education. Current optimization strategies focus on enhancing tumor infiltration, overcoming immunosuppression with “armored” CAR-Ms, and improving safety with suicide switches. Encouraging pre-clinical data accelerates CAR-M into early-phase clinical trials for solid tumors, and the platform’s utility is also being explored beyond oncology in infectious, autoimmune, and neurodegenerative diseases.

## Introduction

Despite significant advancements in diagnosis and treatment, cancer remains a formidable challenge and a global health crisis. Conventional approaches like surgery, radiation, and chemotherapy are often hampered by widespread side effects, drug resistance, and limited effectiveness against advanced or metastatic tumors [1]. This persistent unmet need has spurred a revolution in oncology, the emergence of immunotherapy. The most significant breakthrough in this new era is adoptive cell therapy, exemplified by chimeric antigen receptor T (CAR-T) cell therapy. CAR-T therapy has demonstrated extraordinary success in treating blood cancers, especially B-cell acute lymphoblastic leukemia and diffuse large B-cell lymphoma, achieving high response rates and long-lasting remissions in patients who have not responded to other treatments [ 2, 3] . These achievements have ignited immense enthusiasm for harnessing the power of the immune system to combat cancer.

However, the impressive efficacy of CAR-T cell therapy in liquid tumors has not been consistently replicated in solid tumors, which constitute over 90% of all cancer diagnoses. CAR-T cells face a multitude of complex challenges within the solid tumor microenvironment (TME) [ 4, 5] . Firstly, the dense extracellular matrix (ECM) and irregular blood vessels in solid tumors form physical barriers that hinder CAR-T cells from reaching and infiltrating the tumor’s core [ 4– 6] . Secondly, solid tumors create a highly immunosuppressive TME, filled with inhibitory cytokines like TGF-β and IL-10, immunosuppressive cells such as myeloid-derived suppressor cells (MDSCs), regulatory T cells (Tregs), and M2-polarized tumor-associated macrophages (TAMs), along with metabolic stresses like hypoxia, acidosis, and nutrient scarcity [ 4, 5] . These factors often render CAR-T cells inactive, leading to functional impairment [7]. Additionally, tumor cells frequently exhibit antigen heterogeneity, with varying antigen expression levels that can result in antigen escape and tumor recurrence [ 4, 5, 8] . The shared expression of antigens between tumors and healthy tissues can also cause severe on-target, off-tumor toxicities [ 4, 5] . Moreover, the harsh TME can lead to rapid exhaustion and apoptosis of CAR-T cells, compromising long-term efficacy. These inherent limitations underscore the urgent need for alternative cellular immunotherapy approaches to effectively combat solid tumors [ 4, 5, 8] .

Within the intricate tapestry of the TME, macrophages are frequently the most abundant immune cell population, sometimes accounting for up to 50% of the tumor mass. Their unique characteristics make them prime candidates for solid tumor immunotherapy [ 9– 11] . Firstly, macrophages possess remarkable plasticity, enabling them to adopt diverse functional phenotypes in response to the microenvironment. They can exist on a spectrum between pro-inflammatory (M1-like) and pro-tumorigenic (M2-like) states. While TAMs typically support tumor growth, angiogenesis, and immune evasion, M1-polarized macrophages demonstrate strong anti-tumor activities, such as effective phagocytosis, antigen presentation, and the release of pro-inflammatory cytokines [ 9, 10] . Notably, macrophages excel at infiltrating dense solid tumors due to their natural migratory ability and amoeboid movement, a trait that sets them apart from lymphocytes [12]. Furthermore, their powerful phagocytic system enables them to consume and break down tumor cells, debris, and even other immune cells within the TME [ 9, 10] . These advantages make macrophages a highly promising alternative to CAR-T cells for treating solid tumors [ 13– 15] .

This insight has driven the creation of chimeric antigen receptor-macrophage (CAR-M) therapy [ 14, 15] . CAR-M involves genetically engineering macrophages to express a CAR, which provides them with specific recognition capabilities towards tumor-associated antigens (TAAs) or tumor-specific antigens (TSAs). The primary aim of CAR-M is to leverage macrophages’ natural abilities to not only directly destroy tumor cells but also to reshape the TME, creating a more favorable setting for the body’s own anti-tumor immune responses [16]. This comprehensive review will focus on the intricacies of CAR-M design and engineering, elucidate their multifaceted anti-tumor mechanisms, discuss the current challenges in their application to various tumor types, highlight advanced strategies for optimizing their therapeutic efficacy, and summarize the pivotal pre-clinical and clinical advancements in this rapidly evolving field.

## Comparison of CAR-T and CAR-M

CAR-M offers distinct advantages over CAR-T cells in solid tumor therapy ( Table 1). Unlike CAR-T cells, which face challenges infiltrating dense tumor tissue, CAR-M secretes matrix metalloproteinases (MMPs) to actively break down the ECM, overcoming physical barriers [ 17, 18] . Additionally, CAR-M can convert “cold” tumors into “hot” ones by releasing pro-inflammatory cytokines [19]. As professional antigen-presenting cells, CAR-M also effectively prevents antigen escape by engulfing tumor debris and presenting a wide range of neoantigens to the adaptive immune system, ensuring a robust, polyclonal response even if the targeted antigen is downregulated [ 15, 20] .

**Table:** **Table 1** Comparison of CAR-T and CAR-M

Feature	CAR-T	CAR-M
Cytotoxicity	Perforin, granzymes, IFN-γ	Phagocytosis, pro-inflammatory cytokines
Solid tumor infiltration	Usually poor (chemokine receptor dependent)	Superior (natural response to TME signals)
Persistence	Long-term(months to years)	Moderate (weeks to months)
Cytokine release syndrome (CRS)	High (20%–90%)	Minimal (nearly absent in early trials)
Neurotoxicity	Common and often serious	Minimal (nearly absent in early trials)
Antigen presentation	None	Professional (MHC-I/MHC-II presentation)
TME remodeling	IFN-γ (pro-inflammatory)	Comprehensive (cytokines, ECM degradation)
Primary indication	Hematologic malignancies (high efficacy)	Solid tumors (especially “cold” tumors)
FDA approved	Six products	None

It is important to note that CAR-M is not meant to replace CAR-T cell therapy, instead, it strategically complements T-cell-based approaches by addressing their inherent limitations. While CAR-T cells boast strong proliferative and cytolytic abilities, they require a supportive environment to function optimally. In this regard, CAR-M acts as a trailblazer, preparing the ground for subsequent T-cell therapies. By utilizing CAR-M to foster an inflammatory tumor microenvironment beforehand, and then combining it with CAR-T therapy, we may discover a promising strategy to significantly boost anti-tumor effectiveness.

## Design and Engineering of CAR-M

**Figure 1:**
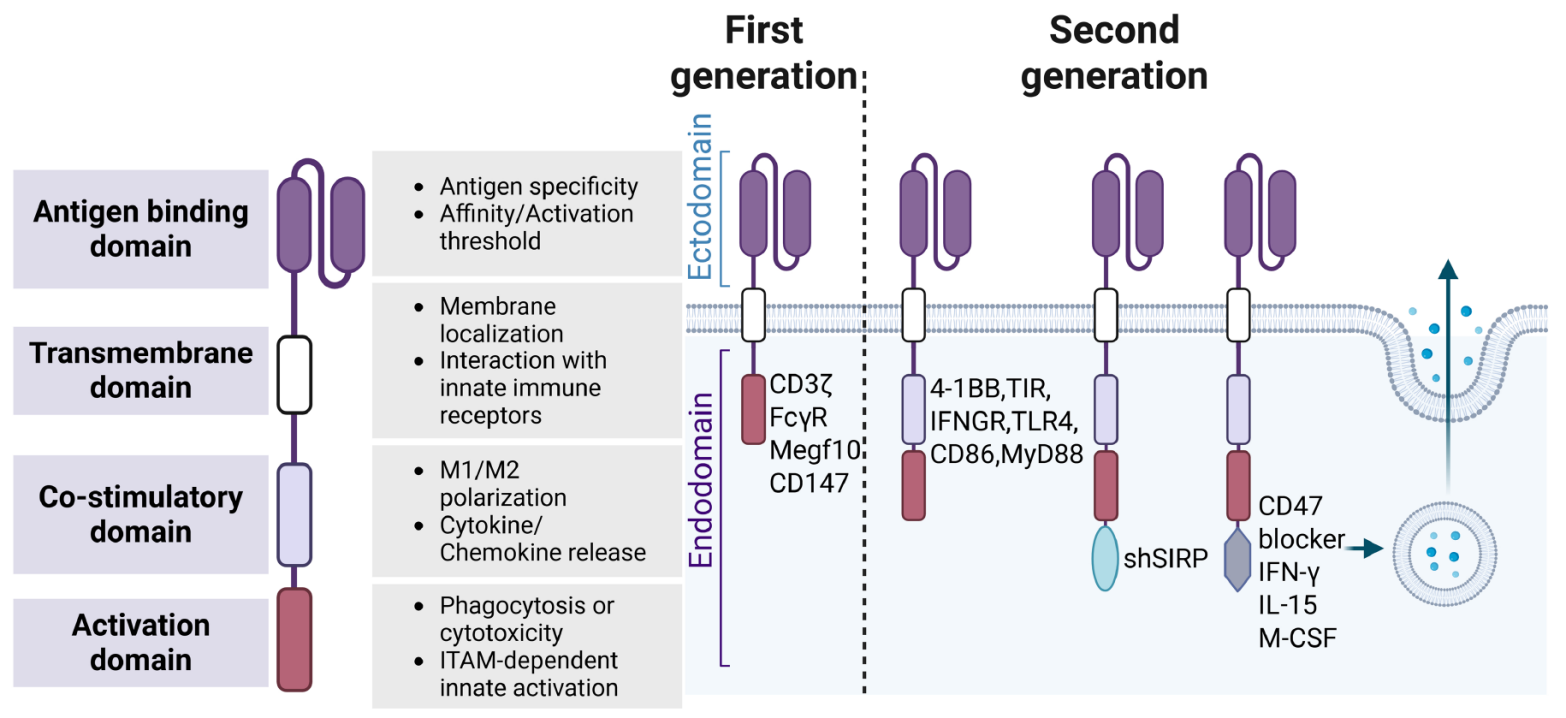
Figure 1 The design of CAR-Ms First-generation CAR-Ms primarily incorporate phagocytosis or innate activation domains, such as CD3ζ, FcγR, Megf10, and CD147, aiming to induce antigen-specific target cell engulfment. Second-generation CAR-Ms further integrate co-stimulatory modules (including 4-1BB, TIR, IFNGR, TLR4, CD86, and MyD88) to enhance inflammatory activation and M1 polarization. Additional functional enhancements, such as PGK promoter-driven shSIRPα for CD47-SIRPα axis blockade or CAR-coupled secretion of soluble CD47 blockers, IFN-γ, IL-15, or M-CSF, expand macrophage effector functions, promote immune-cell recruitment, and improve antitumor activity. Image created with Biorender.com with permission.

The success of CAR-M therapy hinges on the thoughtful design of the CAR construct and the safe, efficient genetic modification of macrophage precursors. CAR, a synthetic receptor, comprises three main parts, an extracellular antigen-binding domain, a transmembrane domain, and an intracellular signaling domain [3]. Each component must be carefully designed for optimal CAR-M performance ( Figure 1).

### Extracellular antigen-binding domain

This domain is typically a single-chain variable fragment (scFv) derived from the variable heavy (VH) and light (VL) chains of a monoclonal antibody, linked by a flexible peptide linker. The scFv confers specificity to the CAR, enabling it to recognize and bind to a specific TAAs/TSAs, expressed on the surface of cancer cells. Choosing the right TAAs/TSAs is crucial, ideal targets should be abundantly and consistently present on tumor cells but rarely on healthy tissues to prevent unintended toxicity [3]. Potential TAAs/TSAs for CAR-M include CD19, CD22, HER2, CEA, EGFRvIII, GPC3, and mesothelin [ 21– 24] . Additionally, the scFv’s affinity for its target can be adjusted to ensure optimal activation without causing constant signaling or immune cell exhaustion [ 25– 27] .

### Transmembrane (TM) domain

This domain serves as a critical structural element that anchors the chimeric receptor, mediates conformational transmission, and facilitates nanoscale clustering through “mechanical coupling”. Variations in the TM’s origin can influence CAR stability on the cell surface, its tendency to dimerize or oligomerize, and its interaction with native receptor complexes, all of which shape activation thresholds and downstream signaling [ 3, 28, 29] . Common TM domains are sourced from CD8α, CD28, CD147, CD4, or CD3ζ [ 17, 22, 30] . The TM’s length and amino acid makeup further dictate how CARs cluster and signal. In macrophage-based CAR platforms, CD8α-derived TM remains the most widely used, typically in combination with a short CD8 hinge, which supports stable receptor surface expression while minimizing Fc-mediated off-target interactions associated with IgG spacers. Clinical-grade HER2-targeted CAR-M constructs like CT-0508 and several pre-clinical studies have adopted this CD8α hinge-TM setup, showing promising expression and anti-tumor effects [31].

### Intracellular signaling domain

This is the most critical component of CAR-M, as it translates antigen binding into an intracellular signaling cascade that dictates the macrophage’s functional output, including phagocytosis, cytokine secretion, and antigen presentation. CAR-M designs have evolved through several generations, primarily differentiated by the complexity of this signaling domain.

#### Intracellular signaling domain in first-generation CAR-M

First-generation CAR-M constructs typically incorporated a single signaling motif for initial activation. Commonly used domains included the cytoplasmic tail of the FcγR, Megf10, MerTK, DAP12, and CD3ζ. CD3ζ, similar to the Fc common γ-chain, triggers antibody-dependent cellular phagocytosis (ADCP) in macrophages [ 32, 33] . CD147 is another key domain, activating MMP expression to break down the ECM and overcome physical barriers [ 17, 34] . While sufficient for initial activation, this single signal often leads to limited functional persistence and anti-tumor activity within the highly suppressive TME.

#### Intracellular signaling domain in second-generation CAR-M

Second-generation CAR-M represents a significant advancement, incorporating one or more costimulatory domains alongside the CD3ζ or FcγR motif. These costimulatory domains provide additional “signal 2” necessary for robust and sustained macrophage activation, analogous to their role in CAR-T cells. Commonly used costimulatory domains include Toll/interleukin-1 receptor (TIR), CD86, interferon-gamma receptor (IFNGR), Toll-like receptor 4 (TLR4), and myeloid differentiation primary response protein 88 (MyD88). Signals from these domains typically trigger rapid and potent activation, leading to early cytokine release and increased metabolic activity [ 21, 30, 35] .

#### Intracellular signaling domain in third-generation CAR-M

A universally accepted definition of “third-generation CAR-M” has yet to be established. However, several emerging designs have functionally surpassed the conventional second-generation structures by incorporating additional functional enhancement modules beyond simple co-stimulatory domains. These advanced designs focus on multidimensional immune regulation. For instance, CAR-Ms with embedded shSIRPα or built-in CD47 blockers can block tumor-derived “don’t eat me” signals, reducing immunosuppression and boosting phagocytosis [ 36, 37] . Another example is CAR-Ms engineered to express membrane-bound IL-15, which actively reshapes the tumor immune environment [38]. Collectively, these functionally augmented CAR-Ms represent a critical shift from simple cellular activation toward multidimensional immune regulation.

## Macrophage Sources and Preparation

The selection of CAR-M sources determines crucial factors for clinical development, including manufacturing scalability, product consistency, potential allergenicity, and the resulting regulatory hurdles.

### Peripheral blood mononuclear cell (PBMC)-derived macrophages

Currently, macrophages derived from PBMCs are the most common source for clinical CAR-M production, as seen with Carisma Therapeutics’ CT-0508 [31]. Monocytes are isolated from a patient’s own blood (autologous) and differentiated *ex vivo* into pro-inflammatory macrophages using granulocyte-macrophage colony-stimulating factor (GM-CSF) [15]. Compared to immortalized cell lines like THP-1 or U-937, PBMC-derived macrophages show stronger inflammatory responses when activated to an M1 state, producing higher levels of cytokines such as IL-6, IL-8, and TNF-α, and displaying markers like CD14 and CD68, indicating a robust innate immune profile [39]. In 2006, Biglari *et al*. [40] first engineered human monocytes with CEA-targeted CARs, proving the therapy’s feasibility and safety. Subsequent studies have demonstrated that CAR-edited PBMC-derived macrophages possess anti-tumor activity [ 15, 31] . This autologous approach avoids immunological rejection, but it is limited by patient-specific cell availability, variability in cell quality, and a relatively high manufacturing cost and time.

### Induced pluripotent stem cell (iPSC)-derived macrophages

Myeloid cells such as macrophages are well known for their limited proliferative capacity. Similar to dendritic cell (DC) therapies, the limited supply of primary cells remains one of the major barriers to the widespread clinical application of macrophage-based therapies. iPSCs represent a transformative source for CAR-M. Unlike primary macrophages, which have restricted proliferation, iPSCs provide a virtually limitless and renewable cell source with consistent differentiation potential [ 41– 43] . This feature greatly improves the engineering efficiency of CAR constructs and enhances batch-to-batch reproducibility, thereby facilitating the development of allogeneic “off-the-shelf” CAR-M products that could significantly reduce manufacturing costs and broaden patient accessibility. Researches has shown that iPSC-derived macrophages share many features with professional phagocytes and demonstrate moderate anti-tumor activity [44], with evidence supporting their effectiveness in leukemia mouse models [45]. Recent studies also indicate that pairing CAR-M therapy with desialylation of cancers enhances anti-tumor immune responses and prevents immune escape [46]. However, its clinical application faces hurdles, including incomplete understanding of their *in vivo* behavior and complex, time-consuming differentiation protocols requiring strict quality control for safety and purity [ 47– 49] . Strategies are underway to genetically modify iPSCs by knocking out MHC-I and MHC-II expression, reducing the risk of immune rejection in allogeneic settings [ 50, 51] .

### Hematopoietic stem and progenitor cells (HSPCs)-derived macrophages

HSPCs are considered a renewable and clinically relevant source for generating macrophages due to their multipotency and long-term reconstitution capacity *in vivo*. HSPCs, typically sourced from cord blood, bone marrow, or peripheral blood, have strong proliferative potential and a primitive phenotype, making them ideal for genome editing [52]. Paasch *et al*. [23] created CAR-Ms from cord blood-derived HSPCs using a lentiviral CAR targeting CEA, showing robust pro-inflammatory responses and tumor-killing ability *in vitro*. Similarly, Zhang *et al*. [43] developed a serum- and feeder-free method to generate CAR-Ms through endothelial-to-hematopoietic transition. Future research should focus on scalable production of HSPC-derived macrophages and explore additional genetic modifications or combinations with other immunotherapies to boost their pro-inflammatory function *in vivo*, which is essential for advancing these therapies into effective models and clinical use.

### Other cell sources

Researchers are also exploring the use of immortalized monocyte cell lines (such as THP-1, Raw264.7, and J774A.1) for pre-clinical studies [50]. While useful for rapid screening, their direct clinical translation is often hampered by safety concerns related to their immortalized nature.

## Efficient and Safe Delivery of CAR

Successfully producing CAR-M hinges on safely and efficiently transferring the CAR gene into macrophage progenitor cells, with lentivirus and adenovirus being the most widely used methods [50].

### Lentiviral vectors

The key advantages of lentiviral vectors include high transduction efficiency, which can efficiently infect both dividing and non-dividing cells; stable genomic integration, the CAR gene is stably integrated into the host cell’s genome, leading to durable and long-term CAR expression in the engineered macrophages. However, bone marrow lineage cells are naturally resistant to HIV-1-based lentiviral vectors due to the myeloid-specific restriction factor SAMHD1, which blocks early viral reverse transcription [ 53, 54] . To overcome this, modified lentiviral vectors have been developed to significantly boost infection efficiency while maintaining therapeutic effectiveness [ 55, 56] . However, lentiviral vectors carry a potential risk of insertional mutagenesis, where the integration of the viral DNA into the host genome could inadvertently activate oncogenes or inactivate tumor suppressor genes. While the risk is generally low, it remains a safety consideration.

### Adenoviral vectors

Adenoviral vectors represent another well-established platform for CAR-M engineering and have attracted growing attention due to their favorable safety and transduction characteristics. Unlike other viral vectors, adenoviruses typically trigger a milder adaptive immune response, indicating lower immunogenicity, and most serotypes remain episomal within infected cell nuclei, reducing the risk of insertional mutagenesis [ 50, 57, 58] . Among these, the chimeric adenovirus vector Ad5/F35, combining elements from adenovirus types 5 and 35, has been shown to improve transduction efficiency in myeloid cells, leading to stronger CAR expression and enhanced anti-tumor activity [59]. Macrophages transduced with Ad5/F35 also tend to adopt a pro-inflammatory state, marked by increased cytokine production and antigen presentation, which may further boost their anti-tumor effects [24]. Additionally, adenoviral vectors have a large packaging capacity (over 8 kb), allowing the delivery of complex CAR constructs with multiple costimulatory domains or regulatory elements in a single unit, offering unique opportunities to fine-tune CAR-M function and optimize its *in vivo* persistence and safety [60]. However, pre-existing immunity to adenoviruses in humans and transient CAR expression remain challenges that require further refinement [15].

### Non-viral methods

Non-viral gene delivery methods, such as cationic polymers and lipid nanoparticles (LNPs), are gaining interest for CAR-M engineering due to their improved safety and potential for rapid, temporary expression [61]. These approaches introduce CAR constructs without integrating into the host genome, avoiding the risk of insertional mutagenesis [62]. The transient expression feature also offers a safety switch, as CAR expressions naturally wane over time, allowing for a more controllable therapeutic window. For clinical manufacturing purposes, mRNA production is generally faster and more scalable than viral vector production. However, mRNA is inherently unstable. The main challenge with mRNA delivery is achieving sustained and high levels of CAR expression. Current research focuses on optimizing mRNA structure and delivery techniques, to enhance stability, translation efficiency, and intracellular delivery, aiming for more sustained CAR expression without genomic integration [ 14, 63– 66] .

## Anti-tumor Mechanisms of CAR-M

**Figure 2:**
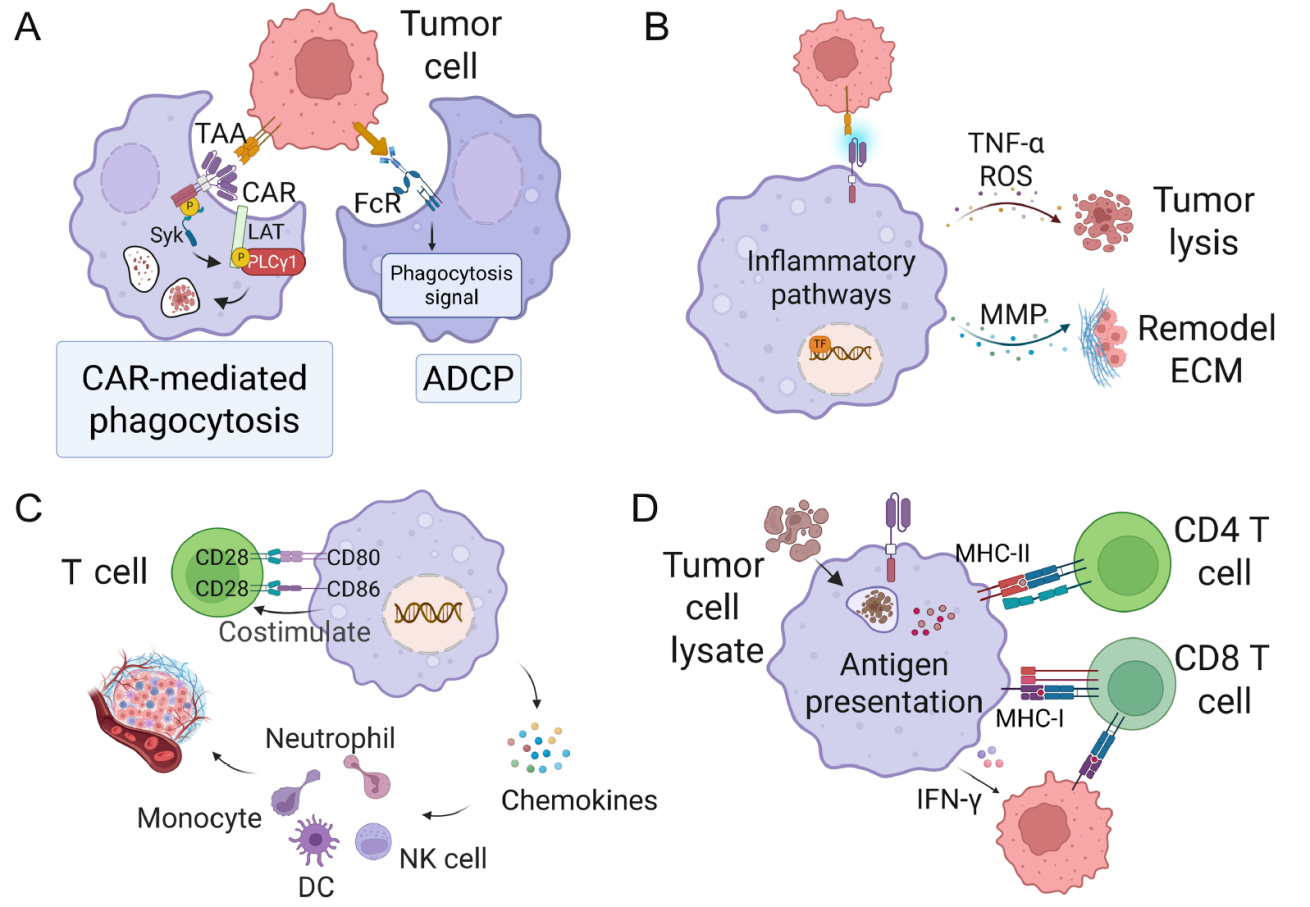
Figure 2 Anti-tumor mechanisms of CAR-Ms (A) Engagement of CAR with TAA triggers Syk-LAT signaling and cytoskeletal rearrangement, leading to direct CAR-driven phagocytosis. In parallel, FcR mediates ADCP of opsonized tumor cells. (B) CAR activation stimulates NF-κB, MAPK, JAK-STAT, and PI3K/AKT signaling, promoting secretion of TNF-α, ROS, and MMPs, which collectively induce tumor cell lysis and remodel the extracellular matrix. (C) CAR-M upregulates co-stimulatory molecules CD80 and CD86 to activate T cells and release chemokines such as CCL2, CCL5, CXCL9, and CXCL10, facilitating recruitment of neutrophils, monocytes, NK cells, and dendritic cells to the tumor microenvironment. (D) Following phagocytosis of tumor debris, CAR-M cells process and present antigens via MHC-I and MHC-II molecules, thereby priming CD8+ and CD4+ T cells and enhancing adaptive anti-tumor immunity. In addition, CAR-M secretes IFN-γ, which upregulates MHC-I/MHC-II expression on tumor cells, thereby enhancing tumor immunogenicity and improving T-cell recognition. Image created with Biorender.com with permission.

By leveraging the targeted accuracy of CARs and the innate cell-destroying capabilities of macrophages, CAR-M therapy employs a synergistic, multi-pronged attack on cancers ( Figure 2)[ 67, 68] .

### Enhanced phagocytosis and direct tumor cell killing: the primary attack

The most direct and immediate anti-tumor mechanism of CAR-M is its antigen-specific phagocytosis. CARs for macrophages typically include ITAM-containing signaling domains like CD3ζ, FcγRs, MerTK, DAP12, CD147, and TLR4 [ 23, 30, 69] . When the CAR binds to a tumor antigen on a tumor cell, it triggers an intracellular signaling cascade within the CAR-M. Src family kinases (Lyn and Fyn) gather at the immune synapse, phosphorylate ITAMs, and recruit SYK and downstream molecules like PI3K and Rho GTPases. This process promotes localized actin polymerization, forming a phagocytic cup [70]. The phagosome then closes and fuses with lysosomes to create a phagolysosome, where hydrolytic enzymes, reactive oxygen species (ROS), and nitric oxide (NO) degrade and destroy the tumor cell [ 22, 71] . Some studies aim to boost the phagocytic activity of CAR-M by combining CAR activation signals with CD47-SIRPα axis blockade, either by blocking CD47 on tumor cells or silencing SIRPα in macrophages [ 36, 37] . This targeted physical destruction of tumor cells is especially valuable in dense solid tumors, where T cell-mediated killing may be less effective [ 72, 73] .

### Tumor microenvironment remodeling: turning the tide

A key strength of CAR-M therapy is its unique ability to reprogram the typically immunosuppressive TME, setting it apart from CAR-T cells, which mainly rely on direct tumor cell killing. This distinguishes CAR-Ms from CAR-T cells, which primarily rely on direct cytotoxicity. In solid tumors, the TME is characterized by a dense infiltration of pro-tumorigenic TAM that exist across a diverse functional continuum, encompassing distinct activation states such as M2a, M2b, M2c, and M2d [74]. These populations collectively suppress cytotoxic immunity, promote immune evasion, and enhance matrix support and angiogenesis to ensure nutrient supply and tumor expansion [75].

CAR-M can mitigate this limitation through multiple strategies. Upon activation via their CAR molecule, CAR-Ms adopt a pro-inflammatory phenotype and actively remodel the surrounding myeloid environment by triggering pro-inflammatory pathways. This paracrine reprogramming helps shift endogenous TAMs from a pro-tumor to an anti-tumor state [ 15, 74, 75] . CAR-Ms secrete powerful anti-tumor mediators, such as TNF-α, IL-1β, IL-6, IL-12, IL-18, and nitric oxide synthase (iNOS) products [ 21, 23, 24] , which amplify local inflammation, induce oxidative stress and apoptotic pathways, promote tumor cell death, and foster a pro-inflammatory Th1 immune response [ 21, 76] . Additionally, CAR-Ms release chemokines like CXCL9/10/11 and CCL5, drawing cytotoxic T lymphocytes (CTLs), DCs, and natural killer (NK) cells into the tumor site, boosting systemic anti-tumor immunity [24]. CAR-Ms also express co-stimulatory molecules CD80 or CD86, enhancing their ability to activate naive T cells. Moreover, activated CAR-Ms can upregulate MHC-I/MHC-II on tumor cells via inflammatory signals like IFN-γ and TNF-α, preventing immune escape and strengthening CD8 ^+^ CTL responses [24]. Macrophages also can secrete enzymes like MMPs that can degrade components of the dense ECM of solid tumors. This matrix remodeling helps physically dismantle barriers, improving the infiltration of CAR-Ms themselves, as well as endogenous T cells and other immune cells, into the tumor core [ 17, 21, 24, 77] . This structural disruption is a key advantage in solid tumors.

### Antigen presentation and adaptive immunity activation: fostering long-term immunity

CAR-M functions as a highly effective antigen-presenting cells (APCs), bridging innate and adaptive immune responses. After internalizing tumor cells through phagocytosis, CAR-M processes the tumor-associated antigens into peptides, which are then loaded onto MHC-I and MHC-II molecules and displayed on the CAR-M surface [ 24, 76] . MHC class I presentation by CAR-M leads to the cross-presentation of tumor antigens to naive and memory CD8 ^+^ CTLs. This can lead to epitope spreading, where immune responses target multiple tumor antigens, making it harder for tumors to escape through antigen loss [ 24, 76] . MHC class II presentation by CAR-M activates CD4 ^+^ helper T cells, which are essential for providing help to CD8 ^+^ T cells, supporting B cell responses, and orchestrating broader anti-tumor immune responses. By efficiently presenting tumor antigens, CAR-M can enhance the anti-tumor immune cascade, fostering a durable and long-lasting response [ 21, 76] .

## Challenges of CAR-M Therapy in Solid Tumors

Solid tumors are the primary focus for CAR-M development, given the persistent limitations of CAR-T cells. However, the complex nature of solid tumors introduces several challenges.

### TME physical barriers

#### Dense ECM

The ECM within the TME is often highly dense and structurally aberrant, forming a formidable physical barrier that impedes infiltration of exogenous immune cells. This dense ECM is mainly produced by cancer-associated fibroblasts (CAFs), which are highly active in the tumor stroma and continuously secrete collagen, fibronectin, hyaluronic acid, and other ECM components [ 78– 80] . CAFs also promote enzyme-mediated crosslinking, which significantly increases ECM stiffness, resulting in a structure with low porosity and high rigidity. This severely restricts CAR-M movement, limiting their ability to penetrate desmoplastic or highly fibrotic tumors [ 80, 81] . To address this, strategies aimed at ECM remodeling, such as inhibiting collagen crosslinking, degrading hyaluronic acid, or deploying localized matrix-degrading enzymes, are emerging as critical approaches to mitigating ECM-mediated barriers, thereby enabling CAR-M therapies to more effectively infiltrate and exert effects within tumor core regions.

#### Aberrant vasculature

Tumor angiogenesis is marked by significant structural and functional irregularities, such as disorganized vessel networks, disrupted endothelial tight junctions, and abnormal vessel permeability [ 82, 83] . These defects weaken vascular stability, leading to poor blood flow and hypoxic areas within the TME. For CAR-Ms, these vascular issues hinder their efficient movement from the bloodstream into the main tumor tissue, causing many transferred cells to become trapped in perivascular or peripheral areas rather than reaching the tumor core [84]. Moreover, hypoxia and nutrient deprivation imposed by aberrant vasculature further diminish CAR-M survival, metabolic fitness, and effector function. Therefore, strategies to “normalize” blood vessels may help restore vascular integrity, improve blood flow, and enhance CAR-M infiltration and long-term function [ 85– 87] .

#### High interstitial pressure

High interstitial fluid pressure (IFP) is a key physical barrier in solid tumors, resulting from abnormal vascular permeability, poor lymphatic drainage, and excessive ECM deposition, which lead to persistent fluid buildup and abnormally high IFP levels [88]. Elevated IFP disrupts fluid exchange between blood vessels and surrounding tissue, significantly reducing the penetration of immune cells, therapies, and large molecules into the tumor core [ 89, 90] . For CAR-Ms, high IFP disrupts chemokine-guided migration, preventing them from reaching deeper tumor areas and promoting an M2-like immunosuppressive state [91]. In addition, sustained mechanical stress negatively impacts cellular viability and metabolism, further compromising CAR-M persistence and therapeutic efficacy. To overcome these issues, combining ECM-degrading enzymes, vascular-normalizing agents, or pressure-reducing physical interventions may help lower IFP and improve CAR-M distribution within the TME [ 92, 93] .

### Immunosuppressive TME

The microenvironment of solid tumors is a master manipulator of immune responses, actively suppressing anti-tumor immunity [ 94, 95] .

#### Immunosuppressive cytokines

Within the TME, a dense network of immunosuppressive cytokines, including TGF-β, IL-10, and prostaglandin E2 (PGE2), functions as a central “brake” on both innate and adaptive immune responses. TGF-β dampens NF-κB/STAT1-driven inflammation through the SMAD pathway, leading to lower expression of MHC-II, CD80/CD86, and IL-12 in macrophages, while boosting tissue repair receptors like CD163 and CD206, promoting an M2-like phenotype [96]. IL-10 strengthens this immunosuppression via STAT3 signaling, further blocking antigen presentation and pro-inflammatory cytokine release, while encouraging immune tolerance [97]. Meanwhile, PGE2 from tumor and stromal cells triggers EP2/EP4-cAMP-PKA signaling, impairing phagocytosis, oxidative burst, and chemotaxis [98]. These soluble factors also enhance “don’t eat me” signals, like CD47/SIRPα and CD24/Siglec-10, making phagocytosis more difficult in cytokine-rich areas [ 99, 100] . For CAR-M, this cytokine milieu not only directly blunts phagocytic and cross-presenting functions but also rapidly “disarms” them upon tumor entry.

#### Metabolic reprogramming

Beyond soluble cytokines, the metabolic architecture of the TME reprograms macrophage function. Hypoxia stabilizes HIF-1α/2α, leading macrophages to upregulate CD39/CD73 and other immunosuppressive molecules, promoting the conversion of ATP to adenosine and increasing VEGF secretion. This deepens their immunosuppressive state [101]. High lactate levels, driven by tumor-produced lactate dehydrogenase A and monocarboxylate transporter 4, along with low extracellular pH, hinder activation of the NLRP3 inflammasome, reduce antigen presentation, and shift macrophages toward fatty acid oxidation-based metabolism, favoring an M2-like state [102]. Additionally, adenosine buildup activates A2A/A2B receptors, triggering cAMP-PKA/CREB signaling that suppresses macrophage, T cell, and NK cell functions [103]. Collectively, this “metabolic landscape” undermines CAR-M survival, motility, antigen presentation, and cytokine secretion, leaving them at a functional disadvantage in hostile tumor ecosystems.

#### Immunosuppressive cell populations

Immunosuppressive cells create strong resistance to CAR-M by outnumbering them, functionally inhibiting their activity, and reshaping the tumor environment. TAMs are key players, displaying remarkable plasticity that allows them to shift between anti-tumor and pro-tumor roles based on local signals [ 104, 105] . In solid tumors, hypoxia and metabolic stress often drive TAMs toward an immunosuppressive state, with their functions varying across different cancers. Furthermore, they also engage in complex interactions with cancer cells, stromal components, and other immune cell populations [106]. These interactions actively support angiogenesis, suppress endogenous immune responses, and remodel the extracellular matrix, thereby facilitating tumor cell invasion and distant metastasis. MDSCs accumulate in response to CSF-1, CCL2/5, and CXCL1/2/5, where they further produce NO and ROS, and eliminate key nutritional factors needed for immune cell proliferation by depleting L-arginine [ 107, 108] . Tregs expand this suppressive circuitry via CTLA-4 engagement, IL-10/TGF-β secretion. CAFs remodel ECM and elevate interstitial fluid pressure, while secreting CXCL12/TGF-β to form physical and chemical “immune suppression zones.”

### Antigen heterogeneity and escape

#### Intratumorally heterogeneity

Unlike many hematological cancers with relatively uniform antigen expressions, solid tumors often display significant heterogeneity in TAA/TSA expressions within different regions of the same tumor or even among individual tumor cells. This can allow “antigen-negative” tumor clones to survive, expand, and trigger relapses [ 109, 110] .

#### Antigen loss/modulation

Tumor cells can evade CAR-M therapy by shedding target antigens or reducing their expression under immune pressure, leading to acquired resistance and immune escape, a major challenge also faced by CAR-T therapy [ 111– 113] .

#### Antigen shedding

Antigen shedding is a biological process in which tumor cells often release surface antigens into the surrounding environment through proteolytic cleavage or other mechanisms. This can significantly affect cancer immunotherapy, especially CAR-T cell therapy [ 114, 115] . Proteolytic cleavage, exocytosis, and factors within the TME are considered major contributors to the process of antigen shedding. Antigen shedding may result in therapeutic resistance, immune modulation, and tumor immune evasion.

### CAR-M polarization, infiltration, and persistence

#### Plasticity as a double-edged sword

While the functional plasticity of macrophages is evolutionarily optimized for tissue homeostasis, it represents a significant intrinsic vulnerability for CAR-M therapy within the immunosuppressive tumor ecosystem [ 116, 117] . Upon infiltrating the tumor, CAR-Ms are immediately subjected to a potent “re-education” program coordinated by the TME. Rather than a simplistic reversal from an M1 to an M2 state, CAR-Ms encounter multi-layered regulatory pressures that can shift their functional identity along a complex, multidimensional polarization continuum [ 11, 74, 75] . Under the chronic influence of tumor-derived metabolites and cytokines, such as lactic acid, TGF-β, and IL-10, the initial CAR-driven transcriptional program may be progressively antagonized. This leads to a phenotypic drift toward a pro-tumorigenic, immunosuppressive state characterized by attenuated phagocytic capacity and impaired antigen presentation [74]. Consequently, another promising dimension of CAR-M therapy involves ensuring the functional persistence of these cells against potent TME-derived polarizing signals, thereby maintaining their activation state and tumor-lytic capacity within a characteristically immunosuppressive microenvironment.

#### CAR-M infiltration

When CAR-Ms are delivered intravenously, they often accumulate less in tumors, as many become trapped in the lungs, liver, and kidneys [118]. In contrast, intraperitoneal administration greatly reduces off-target retention and increases tumor-specific delivery [119].

#### Limited proliferation

Compared with lymphocytes, mature macrophages are terminally differentiated cells and generally have limited proliferative capacity *in vivo*. When CAR-iMac cells were injected into NSG mice, they expanded about two-fold by day 3, persisted for over 20 days, and then gradually declined after around 30 days [44].

## Strategies to Optimize CAR-M Therapy

**Figure 3:**
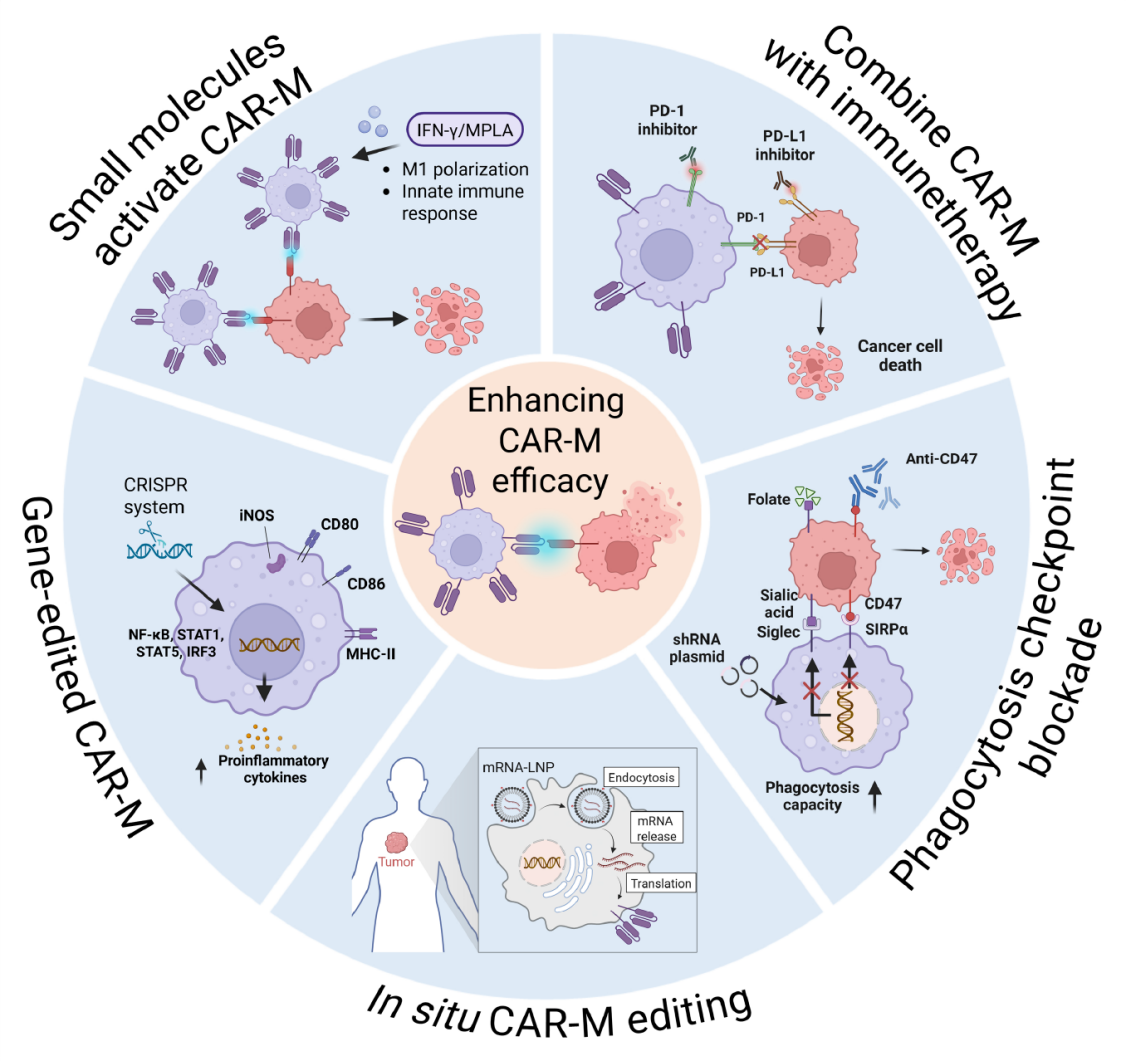
Figure 3 Strategies to optimize CAR-M therapies Researchers are exploring multiple strategies to optimize CAR-M therapy, leveraging diverse mechanisms to potentially enhance its efficacy. Image created with Biorender.com with permission.

Significant efforts are now underway to optimize the design, manufacturing, and deployment of CAR-M ( Figure 3). This optimization is crucial for overcoming existing challenges and translating their immense promise into effective clinical therapies.

### Enhancing CAR-M infiltration and persistence

Although macrophages are easier to infiltrate into TME compared to T cells and NK cells, CAR-M can be further engineered to express enhanced expression of chemokine receptors, such as CCR2, CXCR4, that match chemokines abundantly expressed within the TME. This “homing” approach helps guide CAR-Ms more efficiently to the tumor site, improving their infiltration [ 120– 122] . Additionally, genetically engineering CAR-Ms to secrete ECM-degrading enzymes, such as hyaluronidase or specific MMPs, can help break down the dense extracellular matrix, allowing deeper tumor penetration [17].

For accessible tumors, regional delivery has been shown to markedly improve intratumoral enrichment. For example, intraperitoneal administration in ovarian cancer mouse models reduces off-target organ uptake and enhances tumor-specific accumulation, while direct intratumoral injection allows CAR-M to bypass systemic trafficking barriers and achieve higher local concentrations, as demonstrated in glioblastoma trials ( *e.g*., NCT03500991) [ 36, 123– 125] . Consequently, alternative delivery routes such as intratumoral, intracavitary, or intrathoracic injection are increasingly being considered to maximize local CAR-M density and antitumor efficacy.

Moreover, biomaterial-based strategies have emerged as a powerful approach to augment CAR-M recruitment and persistence *in situ*. Techniques using hydrogels, nanoparticles, or microparticles can reprogram endogenous macrophages directly in the TME, greatly improving their accumulation and infiltration [ 14, 126] . In one study, mesenchymal stem cells delivered CAR-encoding plasmids specifically to glioma-associated macrophages, resulting in stronger intratumoral CAR-M generation than LNP delivery [127]. Similarly, Liu *et al*. [64] developed a light-responsive system using functional liposomes to help macrophages cross the blood-brain barrier and generate CAR-Ms *in situ*.

Providing sufficient long-term cytokine support is essential for the persistence and proliferation of CAR-M. Administering cytokines like GM-CSF, IL-15, IL-12, SCF, and IL-3 alongside CAR-Ms, or genetically modifying them to secrete these cytokines autonomously, has been shown to improve their survival, expansion, and anti-tumor activity *in vivo* [ 35, 128– 130] .

### Overcoming TME immunosuppression

By leveraging their unique ability to remodel the TME, this approach represents a critical optimization strategy for CAR-M. “Armored” CAR-Ms were developed to express and secrete molecules that directly counteract immunosuppression. There are several major types of “armored” CAR-Ms. One type constitutively releases pro-inflammatory cytokines like IL-12, IL-15, or IFN-γ, activating other immune cells, promoting M1 polarization, and sustaining anti-tumor immunity [ 14, 131– 133] . Another type expresses dominant-negative receptors for immunosuppressive cytokines such as TGF-β, effectively blocking suppressive signals within the TME and enhancing therapeutic efficacy [ 134, 135] .

In addition, immune checkpoint blockade has emerged as an important area for CAR-M engineering. Programmed death-1 (PD-1), an immune checkpoint receptor, is markedly upregulated on TAMs, promoting immune tolerance and Treg expansion [ 12, 136] . To counteract this immunosuppressive mechanism, engineered CAR-Ms could be designed to secrete PD-1-blocking scFvs, enabling localized disruption of the PD-1/PD-L1 axis within the tumor microenvironment. This approach may restore cytotoxic T-cell function and enhance antitumor immunity [ 68, 136– 140] . Combination with systemic immune checkpoint inhibitors (ICIs) such as anti-PD-1 or anti-PD-L1 antibodies could represent another promising synergistic strategy. By functioning as professional APCs and reprogramming the TME, CAR-Ms can prime T cells, which can then be further unleashed through ICI therapy, resulting in more potent and durable antitumor immune responses [ 24, 136, 141] .

Another critical immunosuppressive axis in solid tumors is mediated by CD47, a “don’t eat me” signal that enables malignant cells to evade phagocytosis. High levels of CD47 expression and activation of the CD47-SIRPα pathway correlate with poor patient survival. To address this, Chen *et al*. [36] created a CAR-M platform that co-expresses a chimeric receptor with an FcγR signaling domain and a soluble CD47 decoy. This design effectively blocked the CD47-SIRPα axis, boosted phagocytosis even with low antigen levels, and reshaped the TME by promoting infiltration of cytotoxic T cells and inflammatory myeloid cells. Similarly, Zhang *et al*. [37] developed HER2-targeted CAR-Ms with integrated shRNA targeting SIRPα, disrupting CD47-mediated immune evasion and further enhancing macrophage anti-tumor activity.

### Boosting CAR-M anti-tumor activity

Researchers are currently exploring novel costimulatory domains, which may be more effective or specifically tailored to macrophage activation pathways, such as activating Fc receptors, IFNGR, CD86, and TLR4/6 signaling domains [ 21, 22, 45] . Similarly, to address antigen heterogeneity and prevent antigen escape, CAR-Ms can be engineered to express CARs that simultaneously recognize two or more distinct tumor antigens. This broader targeting approach makes it harder for tumor cells to avoid detection by losing just one antigen, enhancing the therapy’s effectiveness [ 14, 68] .

Inspired by CAR-T immunotherapy, gene-editing strategies can be incorporated into CAR-M therapies to achieve more effective treatment outcomes in solid tumors. CRISPR is commonly used to boost macrophage adaptability and function by knocking out inhibitory genes, promoting sustained M1 polarization and tumor rejection [ 141– 144] . Wu *et al*. [46] used CRISPR to delete phagocytosis checkpoints Siglec5/10 in CAR-Ms, enhancing their phagocytic and inflammatory activity. Liu *et al*. [145] conducted CRISPR screens during the co-culture of CAR-Ms with tumor cells, identifying ATG9A in tumor cells as a key regulator of macrophage cytotoxicity, its depletion increased cancer cell susceptibility to macrophage-mediated killing *in vitro* and *in vivo*.

Small-molecule immune modulators can reprogram CAR-Ms toward a pro-inflammatory, tumoricidal phenotype, thereby enhancing their cytotoxicity, delaying tumor progression, and improving survival in preclinical models. For example, IFN-γ and monophosphoryl lipid A have been used to activate innate immunity and reprogram HPSC-derived CAR-Ms into potent tumor-killing macrophages [35]. Similarly, treating CAR-Ms with LPS and IFN-γ *in vitro* effectively inhibited tumor progression, prolonged survival in tumor-bearing mice, and enhanced cytotoxicity [146]. Monoclonal antibodies that trigger ADCP also improve macrophage-mediated tumor killing, while inhibitors targeting CD47/SIRPα signaling have shown promise in boosting macrophage-driven anti-tumor immunity [ 35, 126, 141, 147] . PD-1 checkpoint inhibitors further enhance macrophage phagocytic activity *in vivo*, significantly extending survival in mouse models [ 24, 136, 141] . Combining CAR-Ms with chemotherapy or oncolytic viruses (OVs) improves tumor control [ 119, 148] , OVs selectively infect and lyse tumor cells, releasing antigens and creating a pro-inflammatory environment that CAR-Ms exploit to amplify anti-tumor immunity [ 149– 151] . New strategies also include pairing CAR-T cells and CAR-Ms, with CAR-T cells upregulating CD80/CD86 on CAR-Ms to ensure effective T cell activation [ 35, 76, 152] .

### Improving safety and controllability

Safety is paramount for all cell therapies [50]. Strategies to mitigate potential toxicities, such as severe cytokine release syndrome (CRS) or off-target effects, are crucial [ 68, 153– 157] .

#### Suicide switches

Incorporating inducible suicide systems, such as inducible caspase-9 (iCasp9), truncated epidermal growth factor receptor (EGFRt), or herpes simplex virus thymidine kinase (HSV-tk), into CAR-M constructs enables the rapid elimination of engineered cells in the event of severe, uncontrollable adverse events. Administration of specific activating agents, such as rapamycin dimerizer, can induce dimerization of the caspase-9 suicide switch, leading to swift cell clearance. The latter also includes protease-off switches, in which protease inhibitors prevent degron cleavage to trigger CAR degradation, offer an essential safety mechanism [ 156, 158, 159] .

#### On/Off or dose-adjustable switches

CAR-M systems can be developed in which CAR expression or activity is precisely controlled by external small molecules, exemplified as doxycycline or tamoxifen-inducible systems. This enables fine-tuning of therapeutic effects and the ability to stop treatment if toxicity arises [160]. Physical on-switches, like focused ultrasound that triggers heat shock-induced CAR expression, have also been developed [161].

#### Selection of highly tumor-specific antigens

Carefully selecting highly tumor-specific antigens that are abundantly expressed on tumor cells but minimally or not at all on healthy tissues is essential for minimizing off-tumor, on-target toxicity [162]. Due to the low expression of TAAs in normal tissues, there is a problem of “off-target, tumor-external” toxicity. Using a targeted dual-antigen and logic gate CAR can enhance recognition specificity. For instance, since pancreatic cancer cells often co-express carcinoembryonic antigen and mesothelin, CAR-Ms can be engineered to selectively target malignant cells expressing both antigens, while sparing normal cells that express only one [ 163, 164] .

#### Allogeneic approaches with reduced immunogenicity

For allogeneic CAR-Ms derived from iPSCs, reducing immunogenicity is key to safe “off-the-shelf” therapies. Strategies include knocking out MHC-I/II genes or their regulators, such as β2-microglobulin, CIITA, and overexpressing human leukocyte antigen-E (HLA-E). These approaches lower the risk of graft-versus-host disease (GvHD), making CAR-M therapies safer and more suitable for widespread clinical use [ 165– 167] .

## Pre-clinical Studies and Clinical Trial Progress

The CAR-M field is accelerating quickly through pre-clinical development toward clinical translation. Extensive evidence from both *in vitro* and animal model studies confirms the compelling potential of CAR-Ms in cancer treatment. In a landmark study, Klichinsky *et al*. [15] showed that human primary macrophages engineered with a HER2-targeting CAR successfully engulfed HER2-positive breast and ovarian cancer cells *in vitro*. In xenograft mouse models, the intravenous infusion of CAR-Ms resulted in both significant tumor regression and extended survival. The study provided elegant evidence that CAR-M re-educates the TME by encouraging M1 polarization and enhancing the recruitment of T cells. Pre-clinical studies on mesothelin-targeting CAR-M provide further evidence of their potential, showing effectiveness against mesothelin-expressing tumors [168]. Although solid tumors are the primary focus, CAR-Ms that target CD19 have shown promising results against B-cell leukemias and lymphomas in preclinical models, clearly demonstrating their versatility across different cancer types [ 35, 44, 45, 152] . Furthermore, advanced techniques like imaging and single-cell RNA sequencing have been used to track CAR-M movement and functional changes, providing key insights into these mechanisms [ 15, 45] . The promising pre-clinical data have propelled CAR-M therapy into early-phase clinical trials. As of the current date, the clinical landscape is primarily spearheaded by Carisma Therapeutics, in collaboration with academic centers ( Table 2).

**Table:** **Table 2** CAR-M therapies in pre-clinical and clinical trials

Clinical trial	Product name	Disease	Target antigen	Phase	Status	Cell source	Delivery method	Route of administration	Ref.
NCT04660929	CT-0508 (anti-HER2 CAR-M)	HER2-overexpressing solid tumors	HER2	Phase I	Active, not recruiting	Autologous PBMCs	Adenoviral vector	Intravenous infusion	[169]
NCT06254807	CT-0525 (anti-HER2 CAR-monocyte)	HER2-overexpressing solid tumors	HER2	Phase I	Active, not recruiting	Autologous PBMCs	Adenoviral vector	Intravenous infusion	[170]
NCT06224738	MAC-001 (human anti-HER2 CAR-M)	HER2 ^+^ advanced gastric cancer with peritoneal metastases	HER2	Early Phase I	Not yet recruiting	Autologous PBMCs	Adenoviral vector	Intraperitoneal infusion	[171]
ChiCTR2400080078	Anti-HER2 CAR-M	Relapsed/refractory ovarian cancer with high HER2 expression	HER2	Phase I	Recruiting	Autologous monocyte-derived macrophages	N/A	N/A	[172]
NCT03608618	MCY-M11 (anti-Mesothelin CAR-M)	Advanced ovarian cancer and peritoneal mesothelioma	Mesothelin	Phase I	Terminated	Autologous PBMCs	mRNA	Intraperitoneal infusion	[173]
NCT06562647	SY001 (anti-Mesothelin CAR-M)	MSLN-expressing ovarian cancer or pancreatic cancer	Mesothelin	Phase I	Recruiting	Autologous PBMCs	N/A	Intravenous infusion	[174]
NCT05138458	MT-101 (anti-CD5 CAR-monocyte)	CD5 ^+^ relapsed/refractory T cell lymphoma	CD5	Phase I/II	Suspended	Autologous PBMCs	mRNA	Intravenous infusion	[175]
NCT05969041	MT-302 (anti-TROP2 CAR-M)	Advanced epithelial cancer	TROP2	Phase I	Recruiting	Circulating myeloid cells	mRNA	Intravenous infusion	[176]
NCT06478693	MT-303 (anti-GPC3 CAR-M)	Advanced or metastatic GPC3-expressing cancer	GPC3	Phase I	Recruiting	Myeloid cells	mRNA	Intravenous infusion	[177]

Translational evidence from the first-in-human phase 1 trial of CT-0508 (NCT04660929) reveals a highly favorable safety profile that distinguishes CAR-M from the conventional CAR-T cell paradigm. Unlike the frequent high-grade CRS and neurotoxicity observed in CAR-T therapies, patients receiving CT-0508 experienced no dose-limiting toxicities, with no reported cases of Grade 3 or higher CRS or neurotoxicity [31]. The initial clinical pharmacokinetics of CT-0508 following systemic intravenous infusion demonstrate a distinct distribution pattern where CAR-Ms primarily sequester in the lungs within the first 24 hours before redistributing to the liver and spleen, with peripheral blood persistence typically limited to a median of 7 to 14 days [31]. This window is significantly shorter than the multi-month persistence often observed with CAR-T cells. Although CAR-M infiltration has been confirmed in patient biopsies, the quantitative trafficking remains restricted.

To overcome these delivery barriers, optimizing the route of administration based on tumor localization is a critical translational strategy. Intratumoral injection offers a direct means to bypass pulmonary sequestration and achieve high local effector concentrations, although it is restricted to accessible lesions [ 178– 180] ; intra-arterial perfusion could be leveraged for organ-specific malignancies to increase regional CAR-M density; and intracavitary administration, such as intraperitoneal or intrapleural infusion, presents a superior approach for treating serosal metastases, effectively confining the CAR-M within the tumor-bearing compartment while minimizing systemic off-target risks [ 15, 36, 125] . These optimized delivery methods, combined with the structural advantages of CAR-monocytes, may significantly enhance the homing efficiency and clinical outcomes of CAR-M therapies. Beyond *ex vivo* cell manufacturing, *in situ* CAR-M leverages advanced delivery vehicles, such as LNP-mRNA, AAV, or bio-responsive hydrogel systems, to directly reprogram TAMs into functional CAR-Ms within the tumor microenvironment [ 126, 181, 182] . Its feasibility has been demonstrated in pre-clinical models of melanoma and renal cell carcinoma [ 66, 183] . *In situ* engineering avoids the homing barriers and lung entrapment associated with systemic cell infusion, and it has lower systemic toxicity [ 19, 184, 185] . However, the path to clinical translation still depends on overcoming several key challenges: achieving precise target specificity, maximizing transduction efficiency, and ensuring the persistence of CAR expression, so reprogramming based on transient mRNA may require repeated dosing [ 186, 187] .

### Context-specific vulnerabilities and limited performance

Despite the potential of CAR-M, its efficacy is highly sensitive to the specific tumor landscape. Studies in mouse models have shown that CAR-Ms perform less effectively compared to CAR-T and CAR-NKT cells in treating glioma, with minimal therapeutic benefits and antigen presentation capabilities [ 178, 188] . This divergence highlights several critical weaknesses of the macrophage platform. Unlike T cells, which can respond to low antigen counts, the mechanical initiation of phagocytosis may require a higher threshold of antigen density to trigger the necessary cytoskeletal rearrangements and phagocytic cup formation. In “cold” or highly suppressive environments like glioblastoma, the plasticity of macrophages often becomes a liability, leading to rapid “re-education” into a pro-tumorigenic M2-like state or functional exhaustion before anti-tumor effects can be realized [ 101, 102] . Harsh metabolic landscapes, characterized by high lactate and hypoxia, can suppress the expression of MHC-II and co-stimulatory molecules like CD80/CD86. This effectively serves the bridge to adaptive immunity, neutralizing the CAR-M’s ability to prime endogenous T cell responses.

To maximize clinical success, it is essential to define the boundaries where CAR-M provides a distinct advantage over traditional lymphocytes ( Table 3). The underperformance of CAR-M in certain models should not be viewed as a failure of the platform, but rather as a guide for refining clinical implementation and trial design. Firstly, given that systemic infusion often leads to cell trapping in the lungs or liver, regional administration is critical for desmoplastic tumors to achieve sufficient local cell density [ 179, 180] . Secondly, to maintain an M1 identity in hostile niches, future constructs must integrate modules for cytokine secretion or checkpoint blockade to resist metabolic and inhibitory signals. Last but not least, successful therapy may require combining CAR-M with vascular-normalizing agents or metabolic modulators to “pre-clear” the TME of lactate and hypoxia, ensuring the macrophages remain functional upon entry.

**Table:** **Table 3** Strategic selection between CAR-M and other cellular platforms

TME characteristic	Preferred platform	Rationale
Dense and desmoplastic	CAR-M	Macrophages utilize amoeboid movement and secrete MMPs to penetrate rigid physical barriers that exclude T cells.
Myeloid-rich TMEs	CAR-M	CAR-M can actively reprogram existing suppressive TAMs into pro-inflammatory M1 states through paracrine signaling.
High antigen heterogeneity	CAR-M	By engulfing tumor cells and presenting diverse internal antigens via MHC-I/MHC-II, CAR-M promotes “epitope spreading” to prevent antigen escape.
Highly proliferative tumors	CAR-T/CAR-NK/CAR-NKT	Lymphocytes possess superior inherent proliferative capacity for rapid expansion, whereas mature macrophages are terminally differentiated with limited division.

### Beyond oncology

The rapidly expanding field of CAR-M technology, originally designed for oncology, is now making waves in treating a wide range of non-malignant pathologies [ 189, 190] ( Table 4). Leveraging the inherent plasticity and phagocytic nature of macrophages, these engineered cells offer a multifaceted approach to treating complex diseases. In antimicrobial applications, CAR-Ms can be genetically engineered to target specific pathogens by recognizing their unique markers, ultimately leading to targeted phagocytosis and the release of pro-inflammatory signals [ 65, 191, 192] . This novel strategy offers a promising solution to overcome the challenges posed by traditional antibiotic resistance. In autoimmune contexts, CAR-Ms can be used to deplete pathogenic cells or deposits, with M2-polarized CAR-Ms serving as valuable immunomodulatory agents that help reduce systemic inflammation [193]. The development of “signal-switch” receptors equips these engineered cells with the ability to sense inflammatory signals and respond with anti-inflammatory actions, thus safeguarding vulnerable tissues like the kidneys from long-term damage [194]. Moreover, in various disease models such as liver, lung, and cardiac tissues, CAR-Ms have shown significant potential in promoting tissue remodeling by targeting ECM components and activated fibroblasts [ 195– 199] . For instance, in conditions like intervertebral disc degeneration [200] and Alzheimer’s disease [201], specialized CAR-Ms have exhibited the ability to restore tissue integrity and improve cognitive function by targeting specific disease-related molecules. The evolution of CAR-M therapy from being primarily focused on eradicating cancer cells to regulating immune responses highlights its versatility in addressing infectious, fibrotic, and degenerative disorders through precise phagocytic and immunomodulatory mechanisms. This groundbreaking technology holds immense promise in revolutionizing the treatment landscape for a wide array of diseases.

**Table:** **Table 4** Pre-clinical studies of CAR-M in non-oncological diseases

Disease	Target antigen	Cell source	Gene delivery vector	Administration method	Ref.
COVID-19	SARS-CoV-2 spike protein	THP-1	Lentivirus	N/A	[ 191, 192]
Sepsis	*S*. *aureus* surface protein A (SasA)	*In situ* programming	Macrophage targeting peptide-modified LNP	Tail vein injection	[202]
Periprosthetic joint infection	*S*. *aureus* surface protein A (SasA)	*In situ* programming	Nanoparticle	Coating the surface of a bone implant	[65]
Liver fibrosis	Urokinase plasminogen-activated receptor (uPAR)	BMDM	Adenovirus (Ad5f35)	Tail vein injection	[195]
Cardiac fibrosis	Fibroblast activation protein (FAP)	BMDM	Lentivirus	Tail vein injection	[ 196, 197]
Cardiac fibrosis	Fibroblast activation protein (FAP)	*In situ* programming	LNP	Tail vein injection	[198]
Myocardial ischemia-reperfusion injury	Fibroblast activation protein (FAP)	BMDM	Lentivirus	Tail vein injection	[199]
Alzheimer′s disease	β amyloid	HoxB8-derived macrophage	Retrovirus	Intracranial injection	[201]
Atherosclerosis	CD47	THP-1	Surface-anchored HPβ-CD LNP	N/A	[203]
Adriamycin-induced nephropathy	TNF-α	BMDM	Lentivirus	Tail vein injection	[194]
Intervertebral disc degeneration	Phosphatidylserine (PtdSer) on apoptotic cells	Primary peritoneal macrophages	Lentivirus	Intradiscal delivery via microneedle array	[200]

## Summary and Prospective

CAR-M therapy represents a critical, emerging chapter in cancer immunotherapy, specifically designed to address the significant limitations faced by CAR-T cells in solid tumors. Macrophages are uniquely positioned for this role due to their robust tumor infiltration capacity, powerful phagocytic machinery, and inherent plasticity that allows them to reprogram the immunosuppressive TME. The foundation of CAR-M efficacy lies in the evolution of its design. Constructs have advanced from first-generation single signaling motifs CD3ζ or FcγR to second-generation designs incorporating co-stimulatory domains TIR or MyD88 for enhanced functional persistence and sustained activation. Current emerging designs go beyond co-stimulation, integrating functional enhancements like secreted CD47 blockers, representing a shift toward multidimensional immune regulation. CAR-M exerts its anti-tumor effects through three key mechanisms: (1) direct tumor cell killing *via* antigen-specific phagocytosis; (2) TME remodeling by repolarizing pro-tumorigenic M2-like TAMs into anti-tumorigenic M1-like macrophages, releasing pro-inflammatory cytokines TNFα, or IL-12, and degrading the dense ECM barrier; and (3) activation of adaptive immunity by functioning as APCs, cross-presenting tumor antigens to CD8 ^+^ T cells to promote durable, systemic anti-tumor responses. Despite these advantages, the clinical application of CAR-M faces significant challenges. These include overcoming the TME’s physical barriers and mitigating its profound immunosuppressive signals that can cause CAR-M to revert to an M2-like state or become exhausted. Furthermore, limited cell proliferation and the risk of antigen heterogeneity and escape remain concerns. Significant efforts are underway to optimize CAR-M. Strategies include engineering CAR-M for enhanced tumor infiltration *via* chemokine receptor expression, employing “armored” CAR-Ms that secrete IL-12 or express dominant-negative TGFβ receptors to overcome TME suppression, and leveraging advanced gene-editing or combination therapies with ICIs or OVs to boost anti-tumor activity. Furthermore, the development of iPSC-derived allogeneic CAR-M and the incorporation of suicide or on-off switches are critical for achieving safer, “off-the-shelf” products. The promising pre-clinical data, exemplified by the HER2-targeted CAR-M (CT-0508) that showed tumor regression and TME remodeling *in vivo*, have successfully propelled several constructs into early-phase clinical trials for various solid tumors. Beyond oncology, CAR-M also demonstrates immense potential in treating infectious, autoimmune, and neurodegenerative diseases, underscoring its broad future therapeutic utility. The continued integration of sophisticated engineering, allogeneic sources, and synergistic combination therapies is poised to translate CAR-M’s potential into a transformative clinical reality.
